# Associations between weekend catch-up sleep and health-related quality of life using a generalized additive model and sex and chronotype sub-analysis

**DOI:** 10.1093/sleep/zsaf262

**Published:** 2025-08-28

**Authors:** Jinkyung Oh, Eunmi Kim, Jungsoo Gim, Iksoo Huh

**Affiliations:** College of Nursing, Seoul National University, Seoul, Republic of Korea; Department of Nursing, Seoul National University Hospital, Seoul, Republic of Korea; College of Nursing, Seoul National University, Seoul, Republic of Korea; Department of Nursing, Seoul National University Hospital, Seoul, Republic of Korea; Department of Biomedical Science, Chosun University, Gwangju, Republic of Korea; BK FOUR Department of Integrative Biological Sciences, Graduate School of Chosun University, Gwangju, Republic of Korea; Institute of Well-aging Medicare & CSU G-LAMP Project Group, Chosun University, Gwangju, Republic of Korea; College of Nursing, Seoul National University, Seoul, Republic of Korea; The Research Institute of Nursing Science, Seoul National University, Seoul, Republic of Korea

**Keywords:** weekend catch-up sleep, sleep duration, health-related quality of life, sex, chronotype, generalized additive model

## Abstract

**Study Objectives:**

Weekend catch-up sleep (WCUS) has been identified as a potential compensatory alternative for weekday sleep deprivation. While previous studies have highlighted the positive association between the appropriate duration of WCUS and health-related quality of life (HRQoL), precise estimation has not been much conducted. Moreover, considering that sex and chronotype may specifically influence the association, a more flexible and detailed approach is required.

**Methods:**

We mainly focused on 15 038 healthy adults without severe medical conditions from the 7th (2016–2018) Korea National Health and Nutrition Examination Survey and used a generalized additive model to investigate nonlinear associations between WCUS and HRQoL. In addition, we conducted subgroup analyses by sex and chronotype to understand the associations from various perspectives.

**Results:**

In the results with the total subjects, we found a significant nonlinear association of a supine S-shape. In this association, the range of WCUS that could significantly improve HRQoL was 0.49–2.13 hours (h), and the corresponding odds ratio (OR) was 1.11–1.26. In addition, we conducted subgroup analyses using sex and chronotype. In the sex subgroup analyses, no significant results were observed in males, whereas females showed a significantly nonlinear supine S-shaped association, and the significant range of WCUS was 0.47–2.10 h (OR = 1.14–1.34). After chronotype was additionally considered, only the intermediate-type and evening-type females showed significant nonlinear associations.

**Conclusions:**

Tailored sleep interventions based on individual characteristics and specific WCUS durations may serve as an effective strategy to improve HRQoL.

## Teaser

**Statement of Significance** Previous studies on weekend catch-up sleep (WCUS) and health-related quality of life (HRQoL) have analyzed WCUS as a categorical variable, which restricted the precise estimation of its associations. Therefore, this study treated WCUS as a continuous variable and identified a nonlinear S-shaped association. The optimal WCUS duration for HRQoL improvement was 0.49–2.13 hours (h) in the total subjects. Furthermore, subgroup analyses considering sex and chronotype revealed that WCUS significantly improved HRQoL in females within 0.47 to 2.10 h. Among females, intermediate and evening chronotype individuals showed significant nonlinear associations. These findings emphasize the importance of tailored sleep recommendations based on individual characteristics and more attention on biological and behavioral mechanisms underlying WCUS effects and their long-term health effects.

## Graphical Abstract

**Graphical Abstract:**
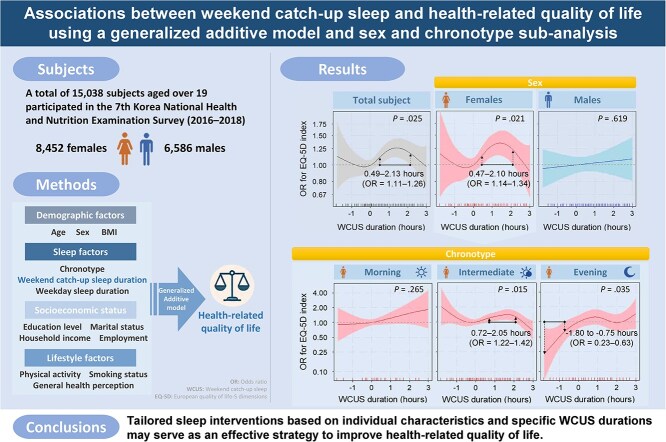

Sleep is an important part of our lives and essential for maintaining physical and mental health. Therefore, healthy sleep habits are closely associated with high quality of our daily lives [1]. On the other hand, both sleep deprivation and excessive sleep are associated with various health issues, including cardiovascular diseases [2], diabetes [3], obesity [4], depression [5], and cognitive impairment [6]. Between the two cases of inappropriate sleep durations, shortened sleep duration becomes more common in modern society due to factors such as extended working hours, shift work, and use of electronic devices [7]. As for this, a study of nearly 9000 United States adults reported that 23.1% of the adult population slept less than the recommended 7 hours (h) on weekdays, and 30.5% of the population experienced a sleep debt of more than 1 h per week, which is defined to be the difference between their actual sleep duration and the recommended sleep duration [8].

In the above cases with problems with shortened sleep duration on weekdays, “weekend catch-up sleep (WCUS),” which implies getting more sleep on the weekends than during the weekdays, has been shown to compensate for the negative effects of sleep deprivation [9]. Considering the difficulties with getting enough sleep during the weekdays for social and economic reasons, WCUS can be a practical and realistic alternative solution to the prevalent sleep deprivation in modern society. Specifically, numerous studies have shown that WCUS can prevent or alleviate metabolic diseases such as obesity [10], hypertension [11], and metabolic syndrome [12, 13], and it can even improve insulin sensitivity [14]. In addition, WCUS has been found to be associated with mental health outcomes, including depression [15], suicidal ideation [16], and anxiety symptoms [17]. To sum up, WCUS is extensively associated with both physical and mental health. In this regard, health-related quality of life (HRQoL), which encompasses a wide range of health conditions in individuals, is also likely to be a candidate variable closely associated with WCUS.

In detail, HRQoL can be defined as the subjective health status that individuals perceive about themselves in physical, mental, and social aspects [18], and consequently it can reflect the complex effects of health on life [19]. Due to these characteristics, HRQoL has been utilized as a key health outcome in clinical fields to assess the effects of chronic diseases or the effectiveness of treatments [20]. Furthermore, it can also be applied to formulate population-level public health policies [21]. Because of the versatile applications of HRQoL, investigating the relationship between HRQoL and more various variables can be encouraged and regarded as an important topic [22], and the interrelationship between sleep and HRQoL also received more attention in recent years [23, 24].

However, relatively little attention has been paid to the association between WCUS and HRQoL, and we could find only two studies [25, 26]. These studies found that WCUS was positively associated with HRQoL. Particularly, Oh et al. [26] recommended WCUS duration over 0 h but under 2 h, and they noted that the effect of WCUS was significantly clear only in females. Considering that several studies have reported a U-shaped or an inverted U-shaped nonlinear association between sleep duration and health [27–29], a simple linear model may also be unsuitable to identify the association between WCUS and health, and applying a nonlinear model is needed to accurately estimate the association. Although in the study of Oh et al. [26], they treated WCUS duration as a discrete dummy variable divided by 1-h intervals, the approach requires an additional assumption that the directions of the association remain consistent within each hour’s interval. In other words, there were limitations in identifying the specific WCUS durations that are more precise than 1-h units. However, no study has yet applied a nonlinear model to examine the relationship between WCUS and HRQoL. Therefore, we aimed to estimate the nonlinear association using a generalized additive model (GAM) in this study.

In addition, we also considered sex and chronotype as key subgroup variables in this study. In the case of sex, previous sleep research has shown that there are sex differences in sleep patterns and qualities due to the influence of sex hormones [26, 30, 31], so it is meaningful to investigate whether there are differences even in the association between WCUS and HRQoL according to sex. Furthermore, chronotype has also been found to be associated with sleep [32, 33], but there is a lack of research examining whether the association of WCUS on HRQoL varies by chronotype. Chronotype is a categorization of a personal tendency about the time for activity and bedtime based on their circadian rhythm repeating every 24 h as a biological cycle [34]. Therefore, it is strongly associated with sleep–wake cycles, cortisol, and melatonin secretion, which are known to significantly influence an individual’s sleep patterns [34]. Accordingly, this study also aimed to more specifically identify the association between WCUS and HRQoL through the subgroup analyses, considering factors such as sex and chronotype.

In summary, we applied a GAM approach not only to the whole dataset, but also to the sub-datasets divided by sex and chronotype, in order to estimate more flexible and precise associations for each dataset, and then interpreted the corresponding physiological mechanisms. We expect that the analysis results from the nonlinear and subgroup approaches can be used as a basis for suggesting tailored sleep durations according to individual characteristics, to more efficiently improve HRQoL.

## Materials and Methods

### Research subjects and materials

The Korea National Health and Nutrition Examination Survey (KNHANES) used in this study is a national cross-sectional survey conducted annually by the Korean Disease Control and Prevention Agency. It conducted a stratified cluster sampling at the household level to reliably represent the population. Within the sampled households, trained surveyors interviewed all eligible household members who were one year old or older. In the interview, they implemented a health screening, health survey, and nutrition survey [35], and the latter two surveys consisted of self-administered questionnaires. Among the survey questions, sleep-related variables and HRQoL were included, so the KNHANES was suitable for this research topic. On the KNHANES website (https://knhanes.kdca.go.kr/knhanes/main.do), anyone can freely download the raw data without any strict approval process. Therefore, this research obtained an exemption from review from the Seoul National University Institutional Review Board (IRB No. E2308/001-003).

**Figure 1:**
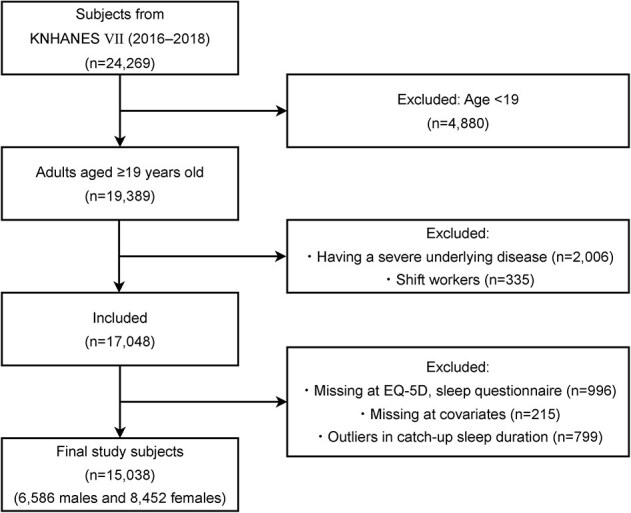
A flow diagram of the filtering process in the research participants. The outliers were defined as the extreme values outside the range of mean ± 2^*^standard deviation.

To be more specific, the subjects in this study were participants in the 7th (2016–2018) KNHANES. Of the total 24 269 subjects who participated in the survey (Figure 1), we excluded 4880 subjects under the age of 19, 2006 subjects with underlying medical conditions that may affect sleep patterns or HRQoL, and 335 shift workers who may have unstable circadian rhythms or sleep patterns [12]. The excluded medical conditions were myocardial infarction, angina pectoris, stroke, rheumatoid and osteoarthritis, cancer, renal failure, cirrhosis, and depression, based on the previous studies [25, 26]. In addition, we excluded 1211 participants with missing values in HRQoL, sleep-related variables, and covariates based on the literature suggesting that imputation of missing values in key variables may bias the results [25]. Finally, we removed 799 outliers whose WCUS duration was outside the mean ± 2*standard deviation range based on previous research that estimated a nonlinear model with sleep duration [28]. Consequently, we included 15 038 individuals in the study, of which 6586 were males and 8452 were females.

In addition, although we primarily focused on healthy adults without severe medical conditions to reduce potential ambiguity and enhance the internal validity of the findings, we also provided additional analysis results that further included participants with the comorbidities in the supplementary material for balanced perspectives. For this analysis, we first grouped these medical conditions into three categories based on their similarities: cardiovascular and cerebrovascular diseases, musculoskeletal and mental disorders, and other chronic diseases and cancers. We then counted the number of comorbidities for each group in each participant as a new covariate. Furthermore, to examine whether the associations varied by age, we also stratified the total participants into non-older (below 65 years) and older adult (65 years or above) groups.

### Sleep-related variables

To generate sleep-related variables, we used responses to the two questions from the KNHANES: “On a weekday (or working day), what time did you go to bed and what time did you get up?” and “On a weekend (or non-working day, the day before a non-working day), what time did you go to bed and what time did you get up?” From the corresponding responses, we calculated weekday and weekend sleep durations, respectively. For WCUS, we used a value that subtracted weekday sleep duration from weekend sleep duration. Based on the definition, a positive value of WCUS for a subject implies that the subject sleeps more on weekends than on weekdays, while a negative value of WCUS has the opposite meaning. All sleep durations were rounded to the third decimal place so that we could represent them to the minute-levels.

Next, for chronotype, we first calculated the mid-sleep time on free days corrected for sleep debt on working days (MSFsc) according to the formula of the Munich Chronotype Questionnaire [36]. Then based on the previous research, we divided the MSFsc values into quintiles and regarded the bottom 20% as morning types, the top 20% as evening types, and the middle 60% as intermediate types [37]. The specific formula for calculating the MSFsc is as follows.


$$ {\boldsymbol{MSF}}_{\boldsymbol{sc}}=\boldsymbol{MSF}-\mathbf{0.5}\times \left[\boldsymbol{SDF}-\frac{\left(\boldsymbol{SDW}\times \mathbf{5}+\boldsymbol{SDF}\times \mathbf{2}\right)}{\mathbf{7}}\right] $$

- MSF (Mid-Sleep on Free days): Median of weekend sleep
- SDF (Sleep Duration on Free days): Sleep duration on weekends
- SDW (Sleep Duration on Workdays): Sleep duration on workdays

### HRQoL

The KNHANES utilizes the European Quality of Life-5 Dimensions-3 Level (EQ-5D-3L), which is a tool to evaluate HRQoL in aspect of the following five subdomains: mobility, self-care, usual activities, pain/discomfort, and anxiety/depression. For each subdomain, response can be one of the three categories (no problem, some problems, or extreme problems). From the responses from the five subdomains, the EQ-5D index is calculated as a single value. In the calculation process, we applied the Korean version of the quality-weighted correction formula [38]. Specifically, if the responses to all of the five sub-domains are “no problem,” the resulting value of the EQ-5D index will be one, which implies that higher scores indicate better HRQoL. However, since the EQ-5D index often exhibits a left-skewed distribution due to the ceiling effect of the instrument itself [39], this study analyzed the EQ-5D index as a dichotomous variable by dividing it into quintiles and grouping them into two groups: one below the first quintile (poor) and the other remaining 80% (not poor), referring to previous studies [26, 40].

### Covariates

Demographic characteristics (age, body mass index [BMI], household income, education, marital status, and employment) and lifestyle habits (smoking, alcohol consumption, and physical activity) were collected through self-administered questionnaires. Among the demographic variables, age was divided into the three categories: younger, middle-aged, and older, at 40 and 65 years based on previous studies concerning HRQoL [41]. BMI was calculated by dividing the body weight by the square of the height. Then we classified BMI into the four categories: underweight (<18.5 kg/m^2^), normal (≥18.5 kg/m^2^, <23.0 kg/m^2^), overweight (≥23.0 kg/m^2^, <25.0 kg/m^2^), and obese (≥25.0 kg/m^2^) [42]. As for the household income, we categorized it into lower, lower middle, upper middle, and upper based on quartiles, and for the education level, we classified the levels into the three groups: middle school or less, high school, and university or higher. We also categorized marital status into the three categories of single, married, and the other (separated, divorced, and widowed), and employment status into dichotomous categories: employed and not employed.

In case of the lifestyle habits, we categorized smoking status into the three groups: never, past, and current smoker. Alcohol consumption states were converted to a dichotomous variable being composed of high-risk drinkers and non-high-risk drinkers. According to the criteria of the KNHANES, we defined the high-risk drinkers as those who consumed an average of seven or more drinks for males and five or more drinks for females each time, along with drinking more than twice a week in the past year [43]. In the KNHANES, physical activity was assessed using the global physical activity questionnaire (GPAQ), and responses to the GPAQ were converted to the metabolic equivalent of task (MET). MET is a quantitative measure of caloric expenditure per unit of body weight at rest, which evaluates physical activity levels. The continuous MET variable was finally classified into the three physical activity levels: low, moderate, and high, following the World Health Organization guidelines [44].

The other covariate is the general health perception, which was selected based on a modified model of HRQoL [45]. According to this model, one of the main factors affecting HRQoL was the subjective perception of health status. The question for the concept was “How do you feel about your health?,” and the response for the question can be one of the five ordinal levels (very poor, poor, fair, good, and very good). Based on previous research, we grouped very poor and poor as “Not good” and the remaining levels as “Good” [25]. Then we used the dichotomized variable as the covariate.

### Statistical analysis

We used R 4.3.1 as the statistical program and the mgcv package in R for GAM analysis. In all of the statistical tests, the significance level was set to be 0.05. The specific methods for the statistical analyses were as follows. First, in the investigation of the general characteristics of the subjects, mean and standard deviation were presented for continuous variables, and frequency and percentage for categorical variables. To compare differences between groups, we applied the Shapiro–Wilk test to check the normality of the continuous dependent variables. When the normality was not satisfied, we applied the Mann–Whitney U-test or the Kruskal–Wallis test. If the dependent variable is categorical, we used the ${\mathrm{\chi}}^2$ independence test. Second, for choosing covariates in the GAM analysis, we fixed the basic demographic variables such as age group, sex, weekday sleep duration, and chronotype in the model. Then we performed the stepwise selection approach and set the *p*-value of .05 as the inclusion and exclusion criteria to construct a model with significant associated explanatory variables and thereby avoid overfitted results [46]. The covariates selected for the total subjects were also used in the sub-analyses by sex and chronotype.

The GAM in the mgcv package utilizes a thin-plate regression spline to estimate nonlinear relationships between variables [47]. The values of the smoothing parameters were estimated using the restricted maximum likelihood method, and the link function was set to be the logit function because the dependent variable is binary. In the GAM plots for visualizing the nonlinear associations between WCUS and HRQoL, we set a point where WCUS duration was 0 h as a base to evaluate odds ratio (OR). Therefore, the corresponding OR in the *y*-axis for WCUS = 0 was set to 1. In addition, the OR curves in the plots were drawn as solid lines, and the regions for 95% confidence interval (CI) of the OR estimates were drawn as the shaded areas surrounding the solid lines.

## Results

### General characteristics of the subjects based on sex and chronotype

Prior to the actual association analysis using GAM, we examined general characteristics of the subjects according to sex and chronotype. When we first divided the total 15 038 subjects by sex, males had significantly higher rates of unhealthy lifestyle behaviors such as smoking and high-risk drinking compared to females (Table 1). Additionally, the proportion of females who were underweight or normal weight was close to half (48.9%), while the proportion of males for the same categories was 32.9%, which indicated that males are relatively heavier than females. However, regarding the general health perception, an effective predictor of HRQoL [45], males (83.7%) responded more to the “good” status than females (79.0%). In a social aspect, the proportion of people who were employed was significantly higher for males (72.4%) than females (51.9%). Next, regarding sleep-related variables, we found that females (7.09 ± 1.34) slept slightly longer during the weekdays than males (7.06 ± 1.27), but this was not statistically significant. However, the WCUS duration of females (0.52 ± 0.92) was significantly longer than males (0.48 ± 0.91). For chronotype, females (66.6%) had a significantly higher proportion of the intermediate type than males (61.4%). Overall, the comparison analysis for the group characteristics showed statistically significant results in all covariates, except for the age variable and the weekday sleep duration.

**Table 1:** Summary of demographic characteristics in the study participants according to sex (*N* = 15 038)

Variables	Categories	Male (*N* = 6586)	Female (*N* = 8452)	*P-value* ^b^
		*n*(%)^a^ or mean ± SD		
Age	Younger	1871 (28.4)	2349 (27.8)	.165
	Middle-aged	3051 (46.3)	4045 (47.9)	
	Older	1664 (25.3)	2058 (24.4)	
Body mass index	Underweight	**149 (2.3)**	**384 (4.5)**	<.001
	Normal weight	**2014 (30.6)**	**3755 (44.4)**	
	Overweight	1686 (25.6)	1742 (20.6)	
	Obesity	2737 (41.6)	2571 (30.4)	
General health perception	Good	**5511 (83.7)**	**6673 (79.0)**	<.001
Education level	$\le$ Middle school	1624 (24.7)	2912 (34.5)	<.001
	High school	2197 (33.4)	2544 (30.1)	
	$\ge$ College	2765 (42.0)	2996 (35.5)	
Household income	Low	1149 (17.5)	1686 (20.0)	<.001
	Low-middle	1583 (24.0)	2078 (24.6)	
	High-middle	1827 (27.7)	2262 (26.8)	
	High	2027 (30.8)	2426 (28.7)	
Marital status	Single	1352 (20.5)	1122 (13.3)	<.001
	Married	4814 (73.1)	5759 (68.1)	
	Etc.^c^	420 (6.4)	1571 (18.6)	
Employment	Employed	**4767 (72.4)**	**4389 (51.9)**	<.001
Physical activity	Low	3540 (53.8)	5023 (59.4)	<.001
	Moderate	2497 (37.9)	3078 (36.4)	
	High	549 (8.3)	351 (4.2)	
Smoking	Never smoker	1569 (23.8)	7546 (89.3)	<.001
	Past smoker	2763 (42.0)	484 (5.7)	
	Current smoker	2254 (34.2)	422 (5.0)	
Drinking	High-risk drinking	1310 (19.9)	448 (5.3)	<.001
Chronotype	Morning	1231 (18.7)	1331 (15.8)	<.001
	Intermediate	**4046 (61.4)**	**5625 (66.6)**	
	Evening	1309 (19.9)	1496 (17.7)	
Weekday sleep duration (h)		**7.06 ± 1.27**	**7.09 ± 1.34**	.058
Weekend catch-up sleep duration (h)		**0.48 ± 0.91**	**0.52 ± 0.92**	.002

Abbreviations: SD, standard deviation.

a The sum of the row percentages may not be 100.0% because of rounding.

b When the normality was not satisfied, Mann–Whitney U-test was performed for the continuous variables. ${\chi}^2$ test was performed for the categorical variables.

c Separated/divorced/widowed.

Next, we divided the total subjects by chronotype and provided the comparison results in Table 2. Among the results, notably, the older adults took the majority in the morning type (63.6%), while the younger adults were the most prevalent in the evening type (63.2%). The dependence between chronotype and age group may also have influenced the relationships between chronotype and some other covariates. For example, the proportion of the highly educated (college degree or higher) was significantly higher in the evening type (47.8%) than in the morning type (11.2%), which may reflect social characteristics associated with the age group. Similarly, the proportion of the subjects with the good general health perception, a job, and a high-middle household income or above was also significantly lower in the morning type than in the evening type. Meanwhile, the proportion of high-risk drinking was significantly higher in the evening type (16.4%) than in the morning (8.6%) and the intermediate type (11.2%). When examining the sleep-related variables, we found a significant decreasing trend in weekday sleep duration in the order of the morning type (7.27 ± 1.44), the intermediate type (7.06 ± 1.25), and the evening type (6.98 ± 1.36). On the other hand, the WCUS duration to compensate for the weekday sleep debt had a significantly increasing trend in the order as above.

**Table 2:** Summary of demographic characteristics in the study participants according to chronotype (*N* = 15 038)

Variables	Categories	Morning (*N* = 2562)	Intermediate (*N* = 9671)	Evening (*N* = 2805)	*P-value* ^b^
		*n* (%)^a^ or mean ± SD			
Age	Younger	70 (2.7)	2376 (24.6)	**1774 (63.2)**	<.001
	Middle-aged	863 (33.7)	5357 (55.4)	876 (31.2)	
	Older	**1629 (63.6)**	1938 (20.0)	155 (5.5)	
Sex	Male	1231 (48.1)	4046 (41.8)	1309 (46.7)	<.001
	Female	1331 (52.0)	5625 (58.2)	1496 (53.3)	
Body mass index	Underweight	64 (2.5)	311 (3.2)	158 (5.6)	<.001
	Normal weight	832 (32.5)	3703 (38.3)	1234 (44.0)	
	Overweight	676 (26.4)	2236 (23.1)	516 (18.4)	
	Obesity	990 (38.6)	3421 (35.4)	897 (32.0)	
General health perception	Good	**1893 (73.9)**	7977 (82.5)	**2314 (82.5)**	<.001
Education level	$\le$ Middle school	1749 (68.3)	2514 (26.0)	273 (9.7)	<.001
	High school	526 (20.5)	3024 (31.3)	1191 (42.5)	
	$\ge$ College	**287 (11.2)**	4133 (42.7)	**1341 (47.8)**	
Household income	Low	995 (38.8)	1489 (15.4)	351 (12.5)	<.001
	Low-middle	747 (29.2)	2274 (23.5)	640 (22.8)	
	High-middle	**458 (17.9)**	2744 (28.4)	**887 (31.6)**	
	High	**362 (14.1)**	3164 (32.7)	**927 (33.1)**	
Marital status	Single	58 (2.3)	1058 (10.9)	1358 (48.4)	<.001
	Married	1880 (73.4)	7427 (76.8)	1266 (45.1)	
	Etc.^c^	624 (24.4)	1186 (12.3)	181 (6.5)	
Employment	Employed	**1272 (49.7)**	6096 (63.0)	**1788 (63.7)**	<.001
Physical activity	Low	1673 (65.3)	5475 (56.6)	1415 (50.5)	<.001
	Moderate	755 (29.5)	3641 (37.7)	1179 (42.0)	
	High	134 (5.2)	555 (5.7)	211 (7.5)	
Smoking	Never smoker	1526 (59.6)	6066 (62.7)	1523 (54.3)	<.001
	Past smoker	732 (28.6)	2027 (21.0)	488 (17.4)	
	Current smoker	304 (11.9)	1578 (16.3)	794 (28.3)	
Drinking	High-risk Drinking	**220 (8.6)**	**1079 (11.2)**	**459 (16.4)**	<.001
Weekday sleep duration (h)		**7.27 ± 1.44**	**7.06 ± 1.25**	**6.98 ± 1.36**	<.001
Weekend catch-up sleep duration (h)		**0.15 ± 0.58**	**0.51 ± 0.91**	**0.80 ± 1.07**	<.001

Abbreviations: SD, standard deviation.

a The sum of the row percentages may not be 100.0% because of rounding.

b When the normality was not satisfied, Mann–Whitney U-test was performed for the continuous variables. ${\chi}^2$ test was performed for the categorical variables.

c Separated/divorced/widowed.

### Association analysis for the total subjects using GAM

As a result of the stepwise variable selection, we removed only the drinking variable from the covariates and included all the other variables in the final covariates. The final selected covariates were age group, sex, BMI, general health perception, education, household income, marital status, employment, physical activity, smoking status, chronotype, and weekday sleep duration, which were presented in Table 3. Then we performed GAM with the WCUS duration and the selected covariates. The results of the GAM analysis are shown in Table 3, which summarizes the statistics and *p*-values of each covariate based on the total subjects. When we interpreted the results based on the regression coefficients, we found that the age variable had a significantly decreasing trend of HRQOL as the age level became high, and males showed a higher HRQoL than females. For BMI, when we arranged the subcategories in ascending order based on HRQoL, the sequence was obese, overweight, underweight, and normal weight. In addition, those who reported a good general health perception, being employed, and higher education or income levels were associated with higher HRQoL than those who did not. Regarding marital status, HRQoL was the lowest in the other group, followed by single, then married. For the covariates about lifestyle habits, the moderate physical activity level and never smokers showed the highest HRQoL, respectively. For chronotype, the subcategories for HRQoL were arranged in ascending order: morning, evening, and intermediate type. In addition to the categorical covariates, the continuous covariate, weekday sleep duration, showed a significant nonlinear association with HRQoL (Effective degree of freedom [EDF] = 3.786, *p* < .001).

**Table 3:** Association analysis results between HRQoL and the independent variables estimated by the generalized additive model for the total participants (*N* = 15 038)

Variables	Categories	Estimate	*P*-value	Overall *P*-value
Age group (ref. younger)	Middle-aged	−0.43	<.001	<.001
	Older	−1.36	<.001	
Sex (ref. male)	Female	−0.35	<.001	
Body mass index (ref. underweight)	Normal weight	0.06	.716	<.001
	Overweight	−0.02	.896	
	Obesity	−0.26	.078	
General health perception (ref. not good)	Good	1.76	<.001	
Education (ref. ≤middle school)	High school	0.53	<.001	<.001
	≥College	0.72	<.001	
Household income (ref. low)	Low-middle	0.37	<.001	<.001
	High-middle	0.45	<.001	
	High	0.45	<.001	
Marital status (ref. single)	Married	0.07	.546	<.001
	Etc.^a^	−0.22	.086	
Employment (ref. not employed)	Employed	0.44	<.001	
Physical activity (ref. low)	Moderate	0.27	<.001	<.001
	High	0.13	.293	
Smoking status (ref. never smoker)	Past smoker	−0.02	.797	.018
	Current smoker	−0.23	.010	
Chronotype (ref. morning)	Intermediate	0.19	.003	.003
	Evening	0.02	.856	
Weekday sleep duration		EDF = 3.786	<.001	
Weekend catch-up sleep duration		EDF = 3.294	.025	

Abbreviations: EDF, effective degree of freedom; HRQoL, health-related quality of life.

This model explained 30.5% of the total variation in the response variable.

a Separated/divorced/widowed

**Figure 2:**
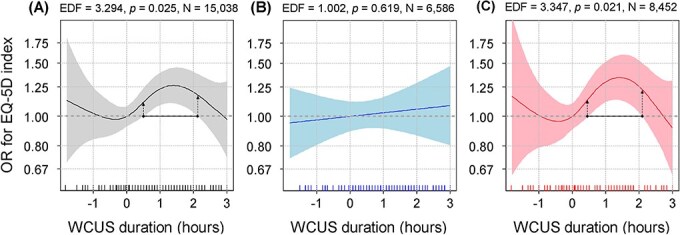
The nonlinear associations between WCUS and HRQoL. (A) Total participants; (B) males; and (C) females. As the EDF becomes closer to one, the pattern of the association becomes more linear.

Next, the variable of interest, WCUS duration, was found to have a significantly nonlinear association in the GAM analysis (EDF = 3.294, *p* = .025). The association plot showed the greatest improvement in HRQoL (OR = 1.26) at 1.40 h of WCUS and the lowest odds of high HRQoL appeared at −0.34 h with an OR of 0.98, compared to 0 h of WCUS (Figure 2, A). The overall nonlinear association showed a supine S-shape. Then we searched intervals where the horizontal line at *y* = 1 did not overlap with the 95% CI of the OR, and we regarded the intervals to have a significantly different OR from 1. In Figure 2, A, we found that the statistically significant interval was 0.49–2.13 h of WCUS (OR = 1.11–1.26), which can be regarded as an optimal range of WCUS duration. Finally, the GAM with the above covariates and WCUS explained 30.5% of the total variation of the response variable (Table 3).

### Subgroup analysis by sex

Subsequently, the results of the GAM analyses in the sex subgroups are shown in Figure 2. For males (Figure 2, B), after adjusting for all covariates selected from the analysis of the total subjects, the WCUS duration was positively and linearly associated with HRQoL but not statistically significant (EDF = 1.002, *p* = .619). In contrast, the results of the GAM analysis in females (Figure 2, C) showed a nonlinear and supine S-shaped association, and it was also statistically significant (EDF = 3.347, *p* = .021). In addition, we found that the optimal range of WCUS duration in females was 0.47–2.10 h (OR = 1.14–1.34).

### Subgroup analysis considering chronotype for each sex

**Figure 3:**
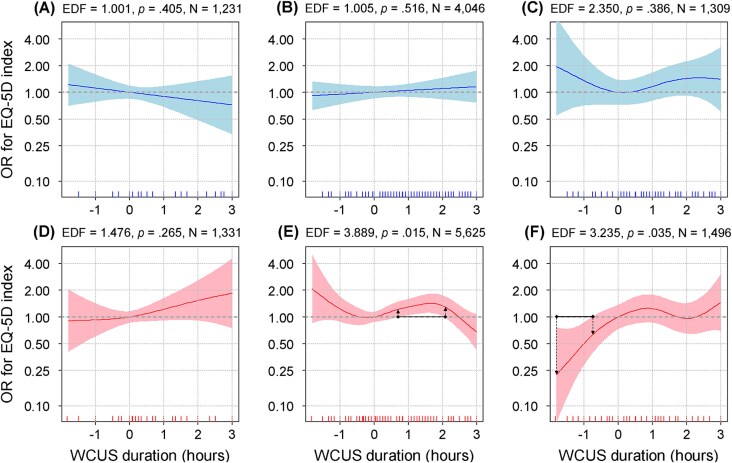
The nonlinear associations between WCUS and HRQOL by sex and chronotype. (A) Morning chronotype males; (B) intermediate chronotype males; (C) evening chronotype males; (D) morning chronotype females; (E) intermediate chronotype females; (F) evening chronotype females. As the EDF becomes closer to one, the pattern of the association becomes more linear.

Next, the results of the GAM analyses by chronotype, in addition to each sex, were presented in Figure 3. In the morning (EDF = 1.001, Figure 3, A) and the intermediate-type males (EDF = 1.005, Figure 3, B), WCUS duration showed an almost linear association with HRQoL, while the evening-type males (EDF = 2.350, Figure 3, C) exhibited a vaguely supine S-shape. However, none of the *p*-values in the three analyses results were significant at the .05 level. In case of females, only the results of the morning-type (EDF = 1.476, Figure 3, D) showed a nearly linear association and statistical insignificance, while the other two types showed nonlinear associations and statistical significance. Specifically, the intermediate type showed a supine S-shaped association (EDF = 3.889, Figure 3, E), and the evening type exhibited a supine inverted S-shaped association (EDF = 3.235, Figure 3, F). Then we investigated the optimal ranges of WCUS duration, which were found only in the intermediate- and the evening-type female subgroup analyses. In detail, the ranges were 0.72–2.05 h (OR = 1.22–1.42) for the intermediate-type females and from −1.80 to −0.75 h (OR = 0.23–0.63) for the evening-type females.

### Investigating influences of extra factors such as age and comorbidities.

When analyzing total participants that further included those with the comorbidities, we observed that the statistical significance slightly decreased, while substantial reductions in both the significance and the nonlinear patterns were observed from the results using only those with the comorbidities (Figures S1 and S2). In the case of the additional analysis with the age stratification, we did not observe much difference in the results of the total and sex subgroup analysis, especially on the overall pattern of nonlinearity and significant intervals (Figure S3). However, when the age stratification was added to sex and chronotype subgroups, we newly observed clear nonlinearities in some of the non-older male subgroups (Figure S4). Specifically, in the non-older males with the morning chronotype, WCUS was associated with reduced HRQoL, while the non-older males with the evening chronotype showed an opposite pattern.

## Discussion

In this study, we investigated the association between WCUS duration and HRQoL in a Korean adult population using GAM, which can detect nonlinear associations between the variables. We also examined whether the association differs by sex and chronotype through subgroup analyses. Methodologically, this study is meaningful in that it is the first one to have shown that WCUS, as a continuous variable, was nonlinearly associated with HRQoL. Furthermore, it has visualized the specific patterns of the associations not only for the total subject, but also for subgroups. Previous studies examining the association between WCUS and HRQoL have treated WCUS as a dichotomous variable [25] or a multinomial variable divided by 1-h intervals [26], so estimation of the nonlinear associations might be restricted in the predefined intervals. On the contrary, by considering WCUS as a continuous variable, this study was able to identify detailed changes in the association across the whole range of the durations, and consequently to provide the ranges of significantly effective WCUS for HRQoL to the minute-level precision. Besides, by defining WCUS to be difference of sleep duration between weekend and weekday, we extended our examination into the part where weekend sleep duration was shorter than weekday sleep duration.

Interpreting our findings based on a clinical aspect, the results of the GAM for the total subjects were consistent with the results in the previous studies about clinically recommended sleep hygiene guidelines. Physiologically, WCUS compensates for sleep debt accumulated during the weekdays [11, 48]. Therefore, excessively short WCUS may not have a meaningful compensatory effect [49]. On the other hand, excessively long WCUS may reduce the exposure to the morning light necessary for advancing the circadian rhythm [50]. Consequently, the resulting delayed circadian rhythm may make hinder waking up on weekdays or workdays [49], and may also interfere with social interactions with friends and family [51]. Therefore, it is advisable to maintain adequate WCUS duration to sufficiently compensate for sleep debts without additional opposite effects. Although there are some differences in dependent variables or subjects, suggested WCUS durations in several studies were also between 0 and 3 h [13, 15, 26].

Next, we examined sex differences in WCUS from the subgroup analysis. In the analysis results, we found that the association of WCUS in females showed significant nonlinearity, while the association in males was almost linear and statistically insignificant. In fact, although results in Table 1 showed that females had slightly longer weekday sleep and WCUS duration than the males, only WCUS duration was significantly different. Considering that previous studies using electroencephalogram (EEG) or actigraphy, together with self-reported questionnaires, found that females generally had longer objective sleep duration than males but tended to report shorter subjective sleep duration [52–55], these differences in our study might also be underestimated. Moreover, previous studies have also reported that longer sleep duration in females could be attributed to physiologically lower sleep quality and efficiency compared to those in males [56, 57]. To put it all together, female subgroup in our data may have substantially longer objective sleep duration and lower sleep quality than the male subgroup, and additional future studies with actigraphy or EEG are needed to confirm it.

In this regard, previous studies have reported that longer sleep duration in females may be attributed to physiologically lower sleep quality and efficiency in females compared to males [56, 57]. In the several studies, females were found to more frequently report sleep difficulties, including increased sleep latency, sleep fragmentation, and decreased sleep quantity than males [31, 56, 58, 59]. These difficulties may be from the physiological differences in sex, because females experience several transitional phases in their life cycle, such as menstruation, pregnancy, childbirth, and menopause. These phases are induced or accompanied by rapid changes in the secretion of sex hormones, and females may also experience undesirable physical symptoms that can disrupt sleep during the processes [60–62]. Concerning this, recent studies have provided a physiological explanation that hormonal changes in females can raise body temperature and thereby disrupt sleep continuity and quality [63, 64]. Moreover, environmental factors such as spousal snoring, children returning home late, and sex role differences in childcare have also been suggested as other factors that interfere with female sleep [65, 66]. Another possible explanation is that females are known to have a higher prevalence of mental health conditions, such as depression and anxiety [67], which could influence HRQoL and the observed associations regardless of sleep patterns.

In addition to these hormonal and environmental factors, previous studies have also reported physiological evidence from sleep EEG. A systematic review of sex-based differences in sleep EEG found moderate evidence that females showed a steeper delta wave slope, whereas males exhibited greater normalized delta power [68]. Delta waves, which occur in the frequency range of 0.5–4 Hz during slow-wave sleep (N3 stage), serve as a key biomarker of sleep homeostasis [69]. Normalized delta power reflects the proportion of slow-wave activity within the total EEG spectrum [70, 71], while a delta wave slope captures the rapidity and synchrony of cortical transitions during deep sleep [72]. Sleep deprivation is known to increase delta wave activity, and its distribution is closely linked to sleep depth, quality, resilience, and need [69]. Taken together, these findings imply that females may achieve more efficient recovery through faster and more synchronized slow-wave activity, while males have an opposite tendency. However, hormonal, environmental, and physiological factors may make women’s sleep more fragmented, potentially increasing vulnerability to weekday sleep debt despite adequate total sleep duration. These sex-specific physiological differences in sleep architecture, such that females may have a greater physiological need for WCUS and more evident compensatory effects of WCUS than males, need to be considered in clinical interventions to sleep-related medical conditions [73].

Consistent with this, previous studies concerning the association between sleep duration and health-related variables have also implicated this physiological need for sleep in females. For example, in a large study with more than 700 000 adults in the United States, Grandner et al. [74] found that the association between short sleep duration and hypertension risk was stronger in females than in males. In addition, Putilov et al. [75], who investigated sleep patterns with 1650 college students, mentioned that female students’ inherent need for longer sleep makes them more vulnerable to weekday sleep deprivation. Therefore, reduced sleep may result in more frequent and harmful negative effects in females.

However, at the same time, some studies have also reported meaningful findings in males. For example, one experimental study found that WCUS significantly improved insulin sensitivity in males [14], and large-sample studies on the effect of WCUS on depression also showed that males exhibited more pronounced benefits than females [76, 77]. These findings suggest that more beneficial sex subgroups from WCUS may vary depending on the types of health-related outcomes. It should also be noted that a lack of statistical significance in male results does not necessarily mean that there is no physiological or clinical effect of WCUS on HRQoL in males. Although the results were not statistically significant, the male total subgroup, as well as the intermediate- and evening-type male subgroups, showed a linearly increasing trend between HRQOL and WCUS. This suggests that if additional appropriate factors, such as social activities, occupation, or age group, are taken into account, the intensity of the association may increase in men. Therefore, further studies with more various explanatory variables and health related outcomes are needed to identify sex differences in sleep and WCUS more clearly.

Finally, in the subgroup analysis considering both sex and chronotype, we found that WCUS was significantly nonlinear only in the intermediate- and the evening-type females. In the intermediate-type females, a range of 0.72 to 2.05 h of WCUS was likely to significantly improve HRQoL, and this range mostly overlapped with the ranges for the total subjects and females. One of the possible interpretations for the similarity is that the characteristics of the intermediate chronotype, which is the most common chronotype (64.3% of the total subjects), were highly reflected in the results of the total subjects. The intermediate chronotype represents a neutral chronotype without a preference for morning or evening [78], and several studies have used the intermediate chronotype as a base group to estimate effects of the morning and the evening chronotypes [79, 80].

However, some other studies have changed perspectives and emphasized the intermediate chronotype. For example, Kim et al. [81] suggested the intermediate chronotype as a protective factor for obstructive sleep apnea, as they found that obstructive sleep apnea was more severe in the morning and evening chronotypes in overweight older adults. In addition, Reiter et al. [82] also mentioned that the circadian rhythm mainly influenced the morning and evening chronotypes, while the homeostatic process mainly influenced the intermediate chronotype. In detail, the two factors in the study stemmed from the two-process model on sleep regulation [83]. Of the two processes, the homeostatic process implies the sleep pressures induced by sleep and wakefulness, while the circadian rhythm is a biological clock which is influenced by external environmental factors such as light. Based on the model, the intermediate chronotype can be regarded to be less affected by environmental factors for the circadian rhythm. In other words, it is more likely to depend on sleep debt and the compensatory effect of WCUS from the homeostatic process than the other two chronotypes. Although the intermediate chronotype has received less attention than the other two chronotypes, it makes up the largest proportion of the population. Therefore, more intensive sleep studies are needed to focus on this chronotype in the future.

In case of the evening-type females, positive WCUS did not significantly improve HRQoL when compared to zero hours of WCUS. However, WCUS less than −0.75 h significantly decreased the OR for HRQoL to 0.63 times. It contrasts the results of the intermediate-type females, where the OR curve in the negative range of WCUS mostly exists over the line of *y* = 1. Considering that the evening chronotype had a significantly shorter weekday sleep duration than the other chronotypes, this finding suggested that late sleep–wake timing with insufficient sleep could not compensate for the sleep debt accumulated during the weekdays. In addition, although not statistically significant, the range of 0 to 2 h of WCUS in the evening-type females also exceeded an OR of 1. Therefore, the trend within the WCUS duration was similar to the results in the total subjects, subgroup analysis for females, and the intermediate-type females.

In the additional analyses regarding comorbidity, we observed substantial reductions in both the significance and the nonlinearities from the results using only those with the comorbidities. These reductions may come from the biological influences of the comorbidities on both sleep quality and HRQoL [24, 84, 85], such as increased inflammation, metabolic dysregulation, or autonomic dysfunction [86, 87]. However, the precise mechanisms underlying these effects require further research.

With respect to age, the age stratification of the total and sex subgroups did not make much difference. Although some studies have reported that appropriate WCUS may have beneficial effects in older adults, such as reducing the risk of cognitive decline [88] and improving HRQoL in older females [26], others have reported limited benefits due to the reduced physical recovery capacity [89]. These inconsistent findings indicate that the underlying physiological mechanisms require more investigation. However, when applying further age stratification to sex and chronotype subgroups, we found that WCUS was negatively associated with HRQoL in the non-older males with the morning chronotype, while it showed a positive association in those with the evening chronotype. These results suggest that the evening chronotype individuals may accumulate greater weekday sleep debt, particularly among non-older adults [90, 91]. This may be due to the circadian misalignment and social jetlag, which result from a mismatch between work and social schedules and biological rhythms, whereas non-older males with the morning chronotype may have more suited circadian rhythms to their social schedules. In such cases, extending sleep on weekends in the morning may disrupt sleep regularity and result in lower HRQoL.

In terms of the strength of this study, this study is meaningful because it is the first study to examine not only the nonlinear association between WCUS and HRQoL, but also the differences in the effects of WCUS according to sex and chronotype. Importantly, this study’s primary outcome, HRQoL, highlighted the multidimensional aspect of perceived health by measuring subjective well-being, which complemented objective health indicators [92, 93]. This approach was meaningful in public health research, where subjective perceptions played a crucial role in health behavior and policy development [94, 95]. In addition, this study also provided specific recommended sleep durations for each subgroup divided by the two characteristics, which can be applied to a tailored sleep intervention. Moreover, since this study found the significant nonlinear associations with WCUS in females, we suggest future studies that focus on more specific populations of females who experience chronic sleep deprivation, such as shift workers.

On the other hand, this study has several limitations. First, because the KNHANES is a cross-sectional data set, the causal inference of the association between WCUS and HRQoL was restricted. Although we attempted to reduce confounding effects by adjusting multiple explanatory variables, it remains unclear whether increased WCUS leads to improved HRQoL, or individuals with better HRQoL are more likely to have catch-up sleep. Therefore, future research may require repeated-measure analyses using day-to-day observations and more objective devices to clarify the directionality and causality. Second, the data from the KNHANES did not include more detailed sleep-related variables such as objective sleep duration, subjective sleep quality, sleep disorders, sleep homeostasis, and napping, which restricted various approaches. Instead, we used weekday sleep duration as a proxy variable for these variables to catch the individual-specific sleep need. However, we acknowledge that this variable has a limited meaning because physiologically required sleep duration can vary among individuals, people who sleep the same hours may have different subjective sleep quality or fatigue. Future research needs to be more multifaceted by combining objective variables measured using actigraphy or EEG with qualitative variables such as subjective sleep quality. Third, the operational definition of chronotype in this study was based on the MCTQ self-report questionnaire, which might differ from objective sleep patterns or chronotype and be influenced by external factors such as psychological or occupational factors [96]. Therefore, the chronotype subgroup findings in this study may be intuitively closer to the self-reported sleep–wake patterns rather than absolute physiological preferences. Future research may need objective chronotype measures, such as actigraphy, melatonin levels, or core body temperature [97]. Fourth, although our study primarily focused on healthy adults without severe comorbidities to enhance internal validity, this approach might reduce the generalizability of the findings and restrict insights about the influence of major diseases on sleep outcomes. Future research with a more specific population may be needed to address this issue. Lastly, HRQoL, as a measure of perceived health status, may not fully correspond to actual health or sleep health status and be influenced by individual perceptions or external factors. Future research may incorporate objective health indicators [19, 94, 95] in addition to HRQoL and consider a wider range of covariates.

## Conclusion

In summary, from the results with the total subjects, we could recommend 0.49 to 2.13 h of WCUS for improving HRQoL. In case of sex subgroup analysis, 0.47 to 2.10 h of WCUS were suggested for the entire female group, while there is no significantly clear suggestion for the entire male group. Considering the chronotype in each sex group, the intermediate-type females showed a significant supine S-shaped pattern similar to the entire female group and the total subjects, and the evening-type females exhibited a significant supine inverted S-shape, while the other subgroup analyses results were not significant. In particular, the evening-type females were associated with a significant decrease in HRQoL when WCUS decreased to less than −0.75 h. This result suggested that the appropriate WCUS duration should be emphasized even more in the evening-type females than in the other chronotypes.

## Supplementary Material

Supplementary_materials_zsaf262

## Data Availability

The original contributions presented in the study are included in the article. Further inquiries can be directed to the corresponding author.
