# Supplementary material for: Associations between weekend catch-up sleep and health-related quality of life using a generalized additive model and sex and chronotype sub-analysis
Source: Sleep. 2025 Aug 28;49(3):zsaf262. doi: 10.1093/sleep/zsaf262 (PMC13016808; doi:10.1093/sleep/zsaf262)
Supplement: Supplementary_materials_zsaf262 [file supplementary_materials_zsaf262.docx]

**Associations between weekend catch-up sleep and health-related quality of life using a generalized additive model and sex and chronotype sub-analysis**

**Jinkyung Oh^a,b^, Eunmi Kim^a,b^, Jungsoo Gim^c,d,e^, Iksoo Huh^a,f*^**

^a^College of Nursing, Seoul National University, Seoul, Republic of Korea

^b^Department of Nursing, Seoul National University Hospital, Seoul, Republic of Korea

^c^Department of Biomedical Science, Chosun University, Gwangju, Republic of Korea

^d^BK FOUR Department of Integrative Biological Sciences, Graduate School of Chosun University, Gwangju, Republic of Korea

^e^Institute of Well-aging Medicare & CSU G-LAMP Project Group, Chosun University, Gwangju, Republic of Korea

^f^The Research Institute of Nursing Science, Seoul National University, Seoul, Republic of Korea

*****Corresponding author:

Iksoo Huh, PhD

Address: College of Nursing; The Research Institute of Nursing Science, Seoul National University, 103, Daehak-ro, Jongno-gu, Seoul, Republic of Korea

E-mail: [huhixoo1@snu.ac.kr](mailto:huhixoo1@snu.ac.kr)

**
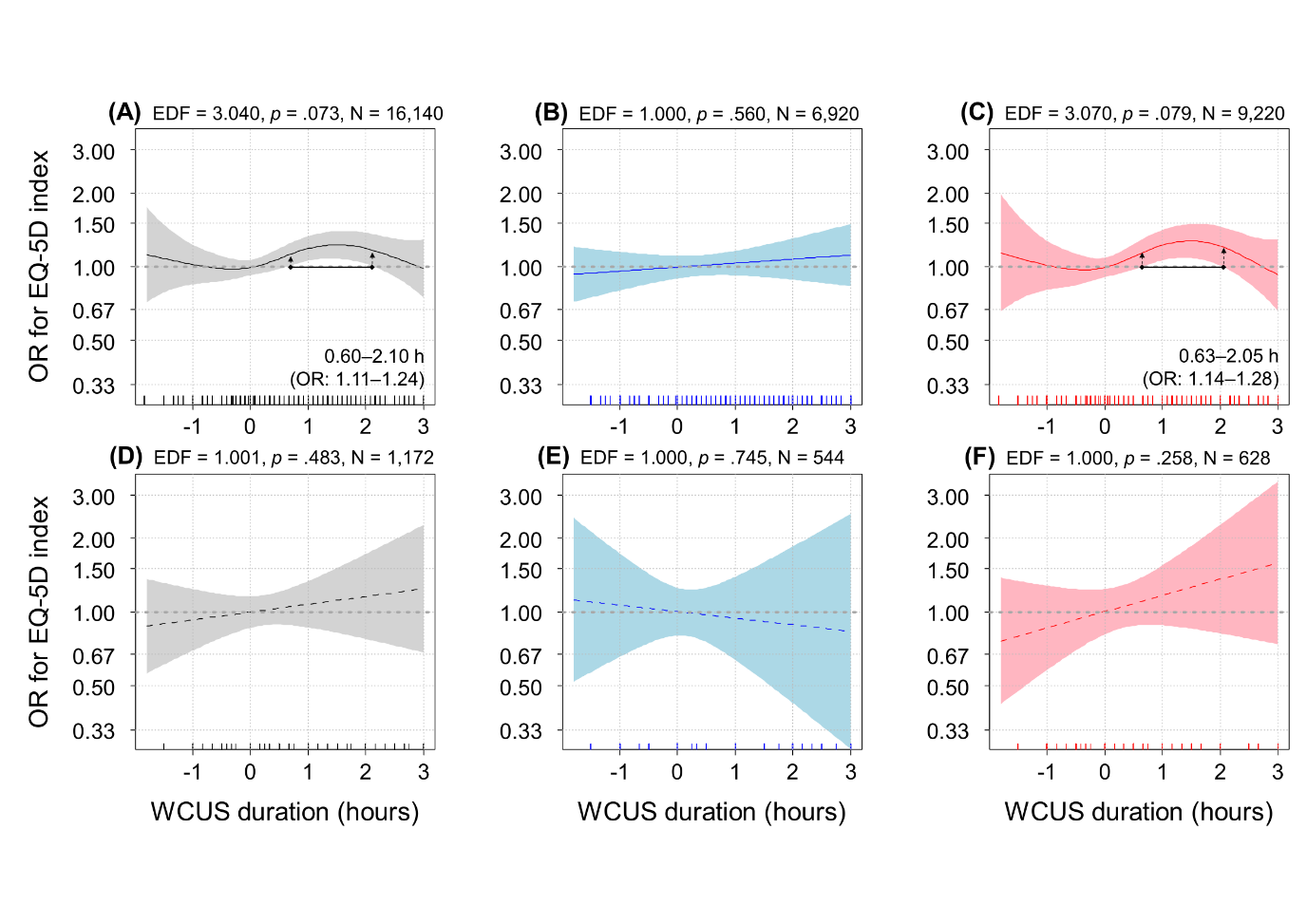
**

**Figure S1. The non-linear associations between WCUS and HRQoL according to comorbidities.** (A–C) All participants regardless of comorbidity status: (A) total participants; (B) males; (C) females. (D–F) Participants with comorbidities only: (D) total participants; (E) males; (F) females.

Abbreviations: EDF, effective degree of freedom; EQ-5D, Euro-quality of life-5 dimension; h, hours; HRQoL, health-related quality of life; OR, odds ratio; WCUS, weekend catch-up sleep.


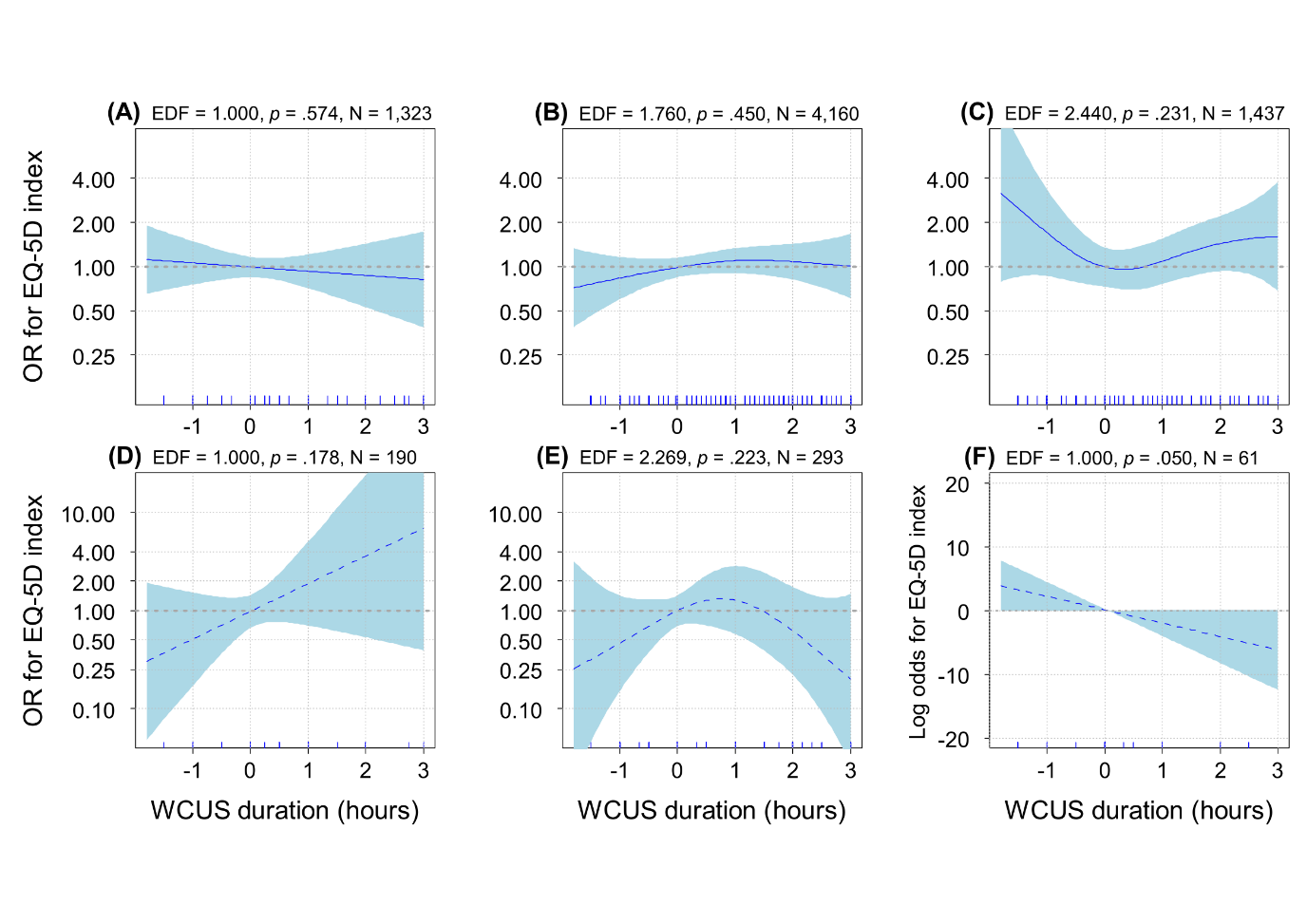

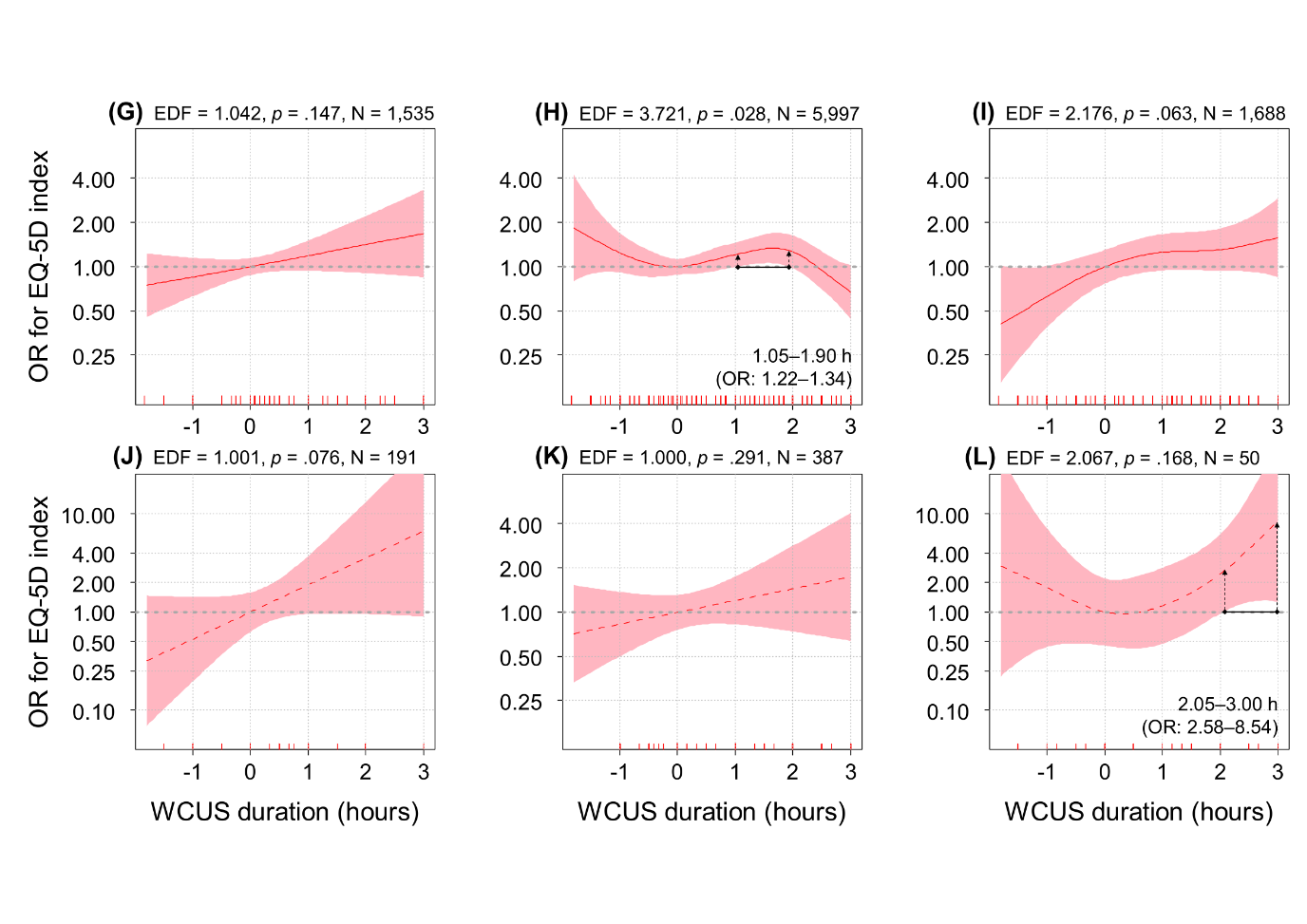


**Figure S2. The non-linear associations between WCUS and HRQoL by sex and chronotype, stratified by comorbidities.** (A–F) Male participants; (G–L) Female participants; (A–C and G–I) Total participants regardless of comorbidity status; (D–F and J-L) comorbidities only; (A, D, G, and J) morning-type; (B, E, H, and K) intermediate-type; (C, F, I, and L) evening-type.

To address failure of model fitting due to small sample sizes in some subgroups (D, F, J, and L), some of the following options were alternatively applied: 1) the bam function, 2) the exclusion of variables with excessively large standard errors, and 3) adjusting k (basis dimension parameter) for WCUS duration according to eigenvalues for model construction. Figure (F) displayed the results with a log-odds scale instead of an odds ratio scale to better visualize the confidence intervals.

Abbreviations: EDF, effective degree of freedom; EQ-5D, Euro-quality of life-5 dimension; h, hours; HRQoL, health-related quality of life; OR, odds ratio; WCUS, weekend catch-up sleep.


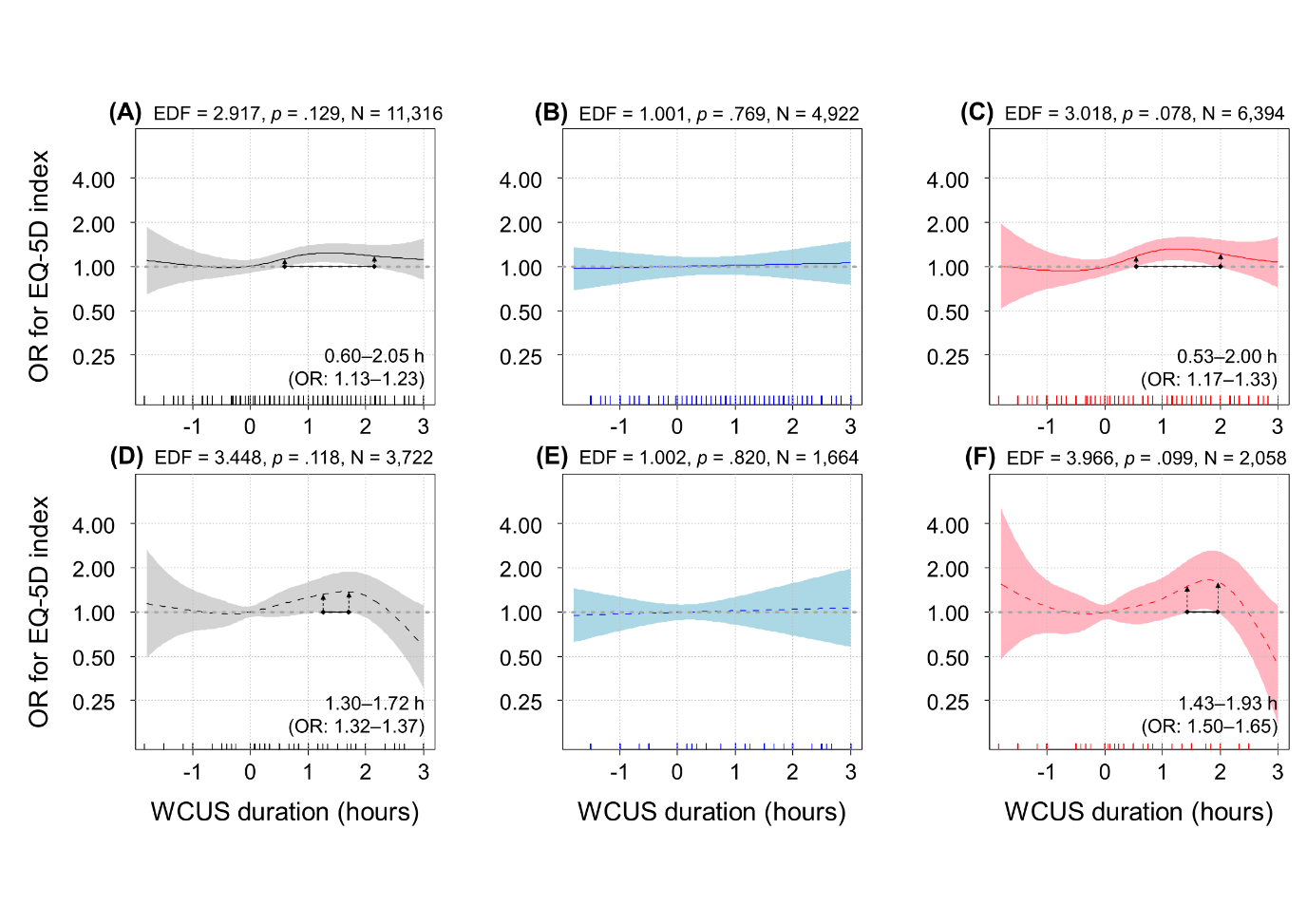


**Figure S3. The non-linear associations between WCUS and HRQoL by age group.** (A–C) Participants aged <65 years: (A) total participants; (B) males; (C) females. (D–F) Participants aged ≥65 years: (D) total participants; (E) males; (F) females.

Abbreviations: EDF, effective degree of freedom; EQ-5D, Euro-quality of life-5 dimension; h, hours; HRQoL, health-related quality of life; OR, odds ratio; WCUS, weekend catch-up sleep.


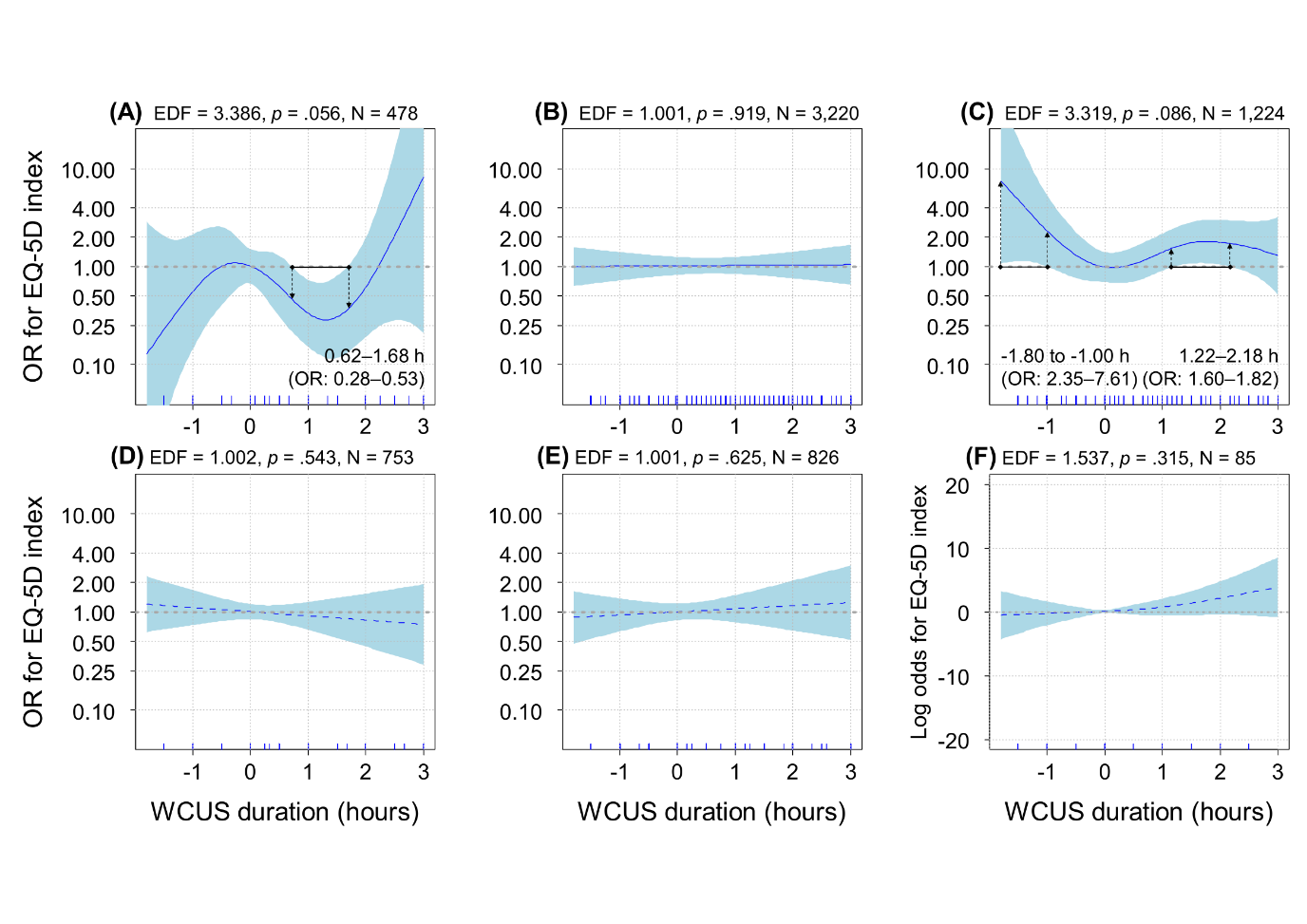

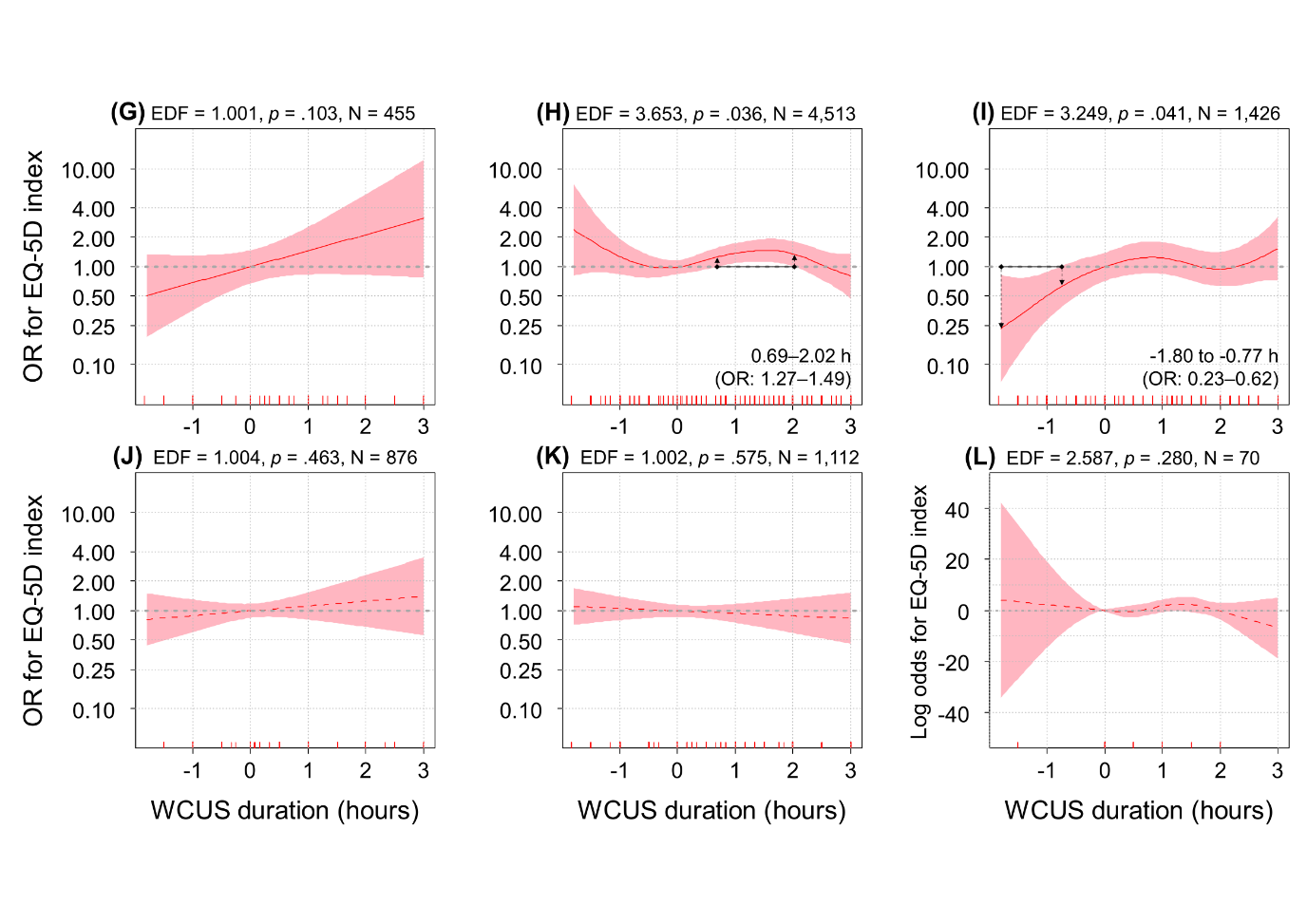


**Figure S4. The non-linear associations between WCUS and HRQOL by sex and chronotype, stratified by age group.** (A–F) Male participants; (G–L) Female participants; (A–C and G–I) Participants aged <65 years; (D–F and J-L) Participants aged ≥65 years; (A, D, G, and J) morning-type; (B, E, H, and K) intermediate-type; (C, F, I, and L) evening-type.

To address failure of model fitting due to small sample sizes in some subgroups (A, D, F, J, and L), some of the following options were alternatively applied: 1) the bam function, 2) the exclusion of variables with excessively large standard errors, 3) adjusting k (basis dimension parameter) for WCUS duration according to eigenvalues for model construction. Figure (F) and (L) displayed the results with a log-odds scale instead of an odds ratio scale to better visualize the confidence intervals.

Abbreviations: EDF, effective degree of freedom; EQ-5D, Euro-quality of life-5 dimension; h, hours; HRQoL, health-related quality of life; OR, odds ratio; WCUS, weekend catch-up sleep.
